# Orthotopic Liver Transplantation in a Patient with Acutely Decompensated Liver Disease and Personal History of Malignant Hyperthermia

**DOI:** 10.1155/2022/4996977

**Published:** 2022-09-17

**Authors:** Elizabeth A. Townsend, Manuchehr Habibi, Molly Groose, Thomas McDowell

**Affiliations:** Department of Anesthesiology, University of Wisconsin School of Medicine and Public Health, Madison, WI 53792, USA

## Abstract

**Introduction:**

Orthotopic liver transplants are characterized by sudden changes in hemodynamics, intraoperative hemorrhage, metabolic and electrolyte derangements, and arrhythmias. Many of these features are also hallmarks of malignant hyperthermia episodes and make differentiation difficult intraoperatively. Additionally, the treatment for malignant hyperthermia, dantrolene, can cause hepatotoxicity in already damaged native livers and newly reperfused organ allografts. Thus, it is imperative to avoid a triggering anesthetic in these patients. Here we report on a successful total intravenous anesthetic in a malignant hyperthermia susceptible individual undergoing an orthotopic liver transplant for acutely decompensated end-stage liver disease. *Case Presentation*. A 49-year-old male with a past medical history significant for malignant hyperthermia episodes as a child was admitted with decompensated alcoholic cirrhosis. He underwent uneventful total intravenous general anesthesia with propofol and sufentanil continuous infusions for an orthotopic liver transplant. He required minimal vasoactive agents to maintain a mean arterial blood pressure >65 mmHg and was extubated on postoperative day 1.

**Conclusions:**

Total intravenous anesthesia is necessary for patients with a personal history of malignant hyperthermia. However, this type of general anesthesia is difficult in the setting of fluctuating hemodynamics, hemorrhage, and changes in drug metabolism and clearance during the anhepatic and reperfusion phases of an orthotopic liver transplant. Propofol and sufentanil continuous infusions provided stable hemodynamics and an excellent plane of anesthesia throughout the case and should be considered in other individuals undergoing this procedure who require a total intravenous anesthetic.

## 1. Introduction

More than 8,000 orthotopic liver transplants (OLTx) are performed in the United States per year and greater than 100 cases are performed at our institution annually. The anesthetic practice for OLTx is not standardized across institutions and relies on provider experience, institutional practices, and patient factors. OLTx is often complicated by intraoperative hemorrhage, sudden changes in preload with caval clamping, alterations in drug metabolism and clearance during the anhepatic and neohepatic phase, and the potential for the postreperfusion syndrome. These factors have either been shown to or have the potential to significantly alter the pharmacokinetics and pharmacodynamics of drugs used for total intravenous anesthesia (TIVA), making volatile anesthetics the preferred agents for anesthetic management for OLTx cases at our institution. TIVAs rely on adequate blood concentrations of anesthetic agents, most commonly opioids and propofol, to maintain unconsciousness and anesthesia; however, monitoring the depth of anesthesia for TIVA is not as straightforward as monitoring minimal alveolar concentration and end-tidal volatile agent concentrations. Despite these disadvantages, patient factors may necessitate the avoidance of volatile agents, specifically in the case of patients with a personal or family history of malignant hyperthermia. Here we review the current literature on liver transplantation surgery in individuals with malignant hyperthermia and present a case of TIVA for OLTx in a patient with acutely decompensated liver disease and a personal history of malignant hyperthermia triggering event as a child during a tonsillectomy.

## 2. Case Presentation

Our patient is a 49-year-old male with a past medical history significant for obesity (BMI 39), hypertension, insulin-dependent type 2 diabetes mellitus, hypothyroidism, previous history of malignant hyperthermia (MH) with tonsillectomy as a child, and a history of end-stage liver disease secondary to alcohol abuse. He was admitted to the hospital 12 days prior to orthotopic liver transplantation for acute decompensation of his liver disease with hepatic encephalopathy and acute kidney injury necessitating continuous renal replacement therapy (CRRT). His model for end-stage liver disease (MELD) at the time of admission was 41. He was approved for United Network for Organ Sharing (UNOS) waitlist for a liver transplant at a multi-disciplinary conference 6 days following his admission. After approval, he suffered a hypoxic respiratory arrest necessitating advanced cardiac life support with the return of spontaneous circulation after 10 minutes of CPR and medical therapy, including intubation. A head CT was completed 2 days following his arrest without evidence of acute intracranial abnormality, and his mental status returned to his baseline prior to his arrest, which was moderately impaired secondary to hepatic encephalopathy.

Following approval for the UNOS waitlist, the anesthesia team discussed the patient's MH history with his spouse. She reported a history of masseter muscle rigidity, elevated temperature, tachycardia, and intensive care unit (ICU) stay related to anesthesia following a tonsillectomy when he was a child. She used specific words including “malignant hyperthermia” and “triggering agent” unprompted when providing anesthesia relevant history. Taken together, the likelihood that this patient experienced a malignant hyperthermia episode while under general anesthesia as a child was high and he was presumed to have malignant hyperthermia in the absence of confirmatory muscle biopsy.

A suitable organ offer became available 11 days after his admission. The patient remained intubated and encephalopathic on CRRT with a MELD of 41 prior to arrival in the OR. At the time of transport to the operating room, the patient was sedated on a continuous infusion of propofol at 30 mcg/kg/min and fentanyl at 25 mcg/hr secondary to endotracheal intubation. He required 0.04 mcg/kg/min of norepinephrine to maintain mean arterial pressure (MAP) > 60 mmHg. Prior to arrival to the OR, the anesthesia machine had been adequately flushed, charcoal filters had been placed, the breathing circuit and carbon dioxide (CO_2_) absorbent canister replaced, volatile agent vaporizers removed from the room, succinylcholine removed from the drug tray, and MH emergency box complete with dantrolene was present in the OR. A bispectral index monitor (BIS) along with standard American Society of Anesthesiologists (ASA) monitors were placed immediately upon arrival at the OR. BIS numbers ranged from 63–68 prior to induction of general anesthesia. Anesthesia was induced with 50 mg of propofol followed by continuous infusion at 50 mcg/kg/min and the addition of continuous sufentanil infusion at 0.2 mcg/kg/hr. The patient's BIS dropped appropriately to 24–40 following induction of anesthesia and remained between 20 and 60 throughout the remainder of the case. In addition to standard ASA monitors and BIS, invasive radial arterial blood pressure monitoring, central venous access with central venous pressure monitoring, and transesophageal echo probe were placed during the procedure prior to incision, and CRRT was continued intraoperatively. For maintenance of anesthesia, the propofol and sufentanil infusions were titrated to maintain a BIS <60 with ranges in propofol from 30 mcg/kg/min to 70 mcg/kg/min and sufentanil ranging from 0.2 mcg/kg/hr to 0.5 mcg/kg/hr. Intermittent boluses of rocuronium bromide were administered to maintain adequate relaxation during the dissection phase with the last dose administered 54 minutes prior to inferior vena cava clamping and the anhepatic phase.

**Figure 1:**
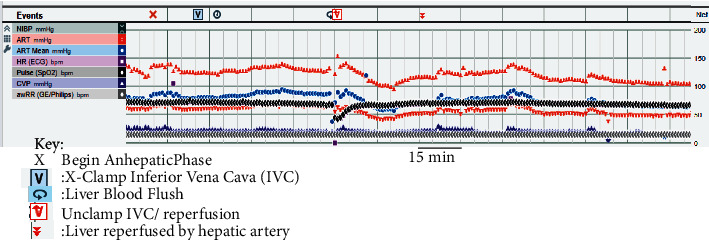
Intraoperative record during anhepatic and reperfusion phases. The patient exhibited marked hemodynamically stability through inferior vena cava cross clamping, anhepatic phase, and reperfusion while under TIVA general anesthetic. Key phases in the intraoperative course are described in the key. Invasive arterial blood pressure (ART; red symbols), heart rate (HR; black diamonds), central venous pressure (CVP; dark blue symbols), mean arterial pressure (ART Mean; light blue circles), and respiratory rate (awRR; grey diamonds). The dark vertical bars represent 15 minute intervals.

The patient tolerated the procedure well without evidence of a malignant hyperthermia episode. Additionally, the patient was very hemodynamically stable throughout the operation and required minimal blood products and vasoactive agents (Table 1 and Figure 1). The sufentanil infusion was discontinued 90 minutes prior to leaving the operating room. The patient remained intubated following the procedure on 30 mcg/kg/min of propofol and returned to the ICU. He was weaned off norepinephrine following his return to the ICU and was extubated on postoperative day 1. Four weeks after OLTx, the patient was interviewed. He had no recall of the time leading up to his procedure or intra-operative awareness.

## 3. Discussion and Conclusions

Malignant hyperthermia is a genetically inherited disorder of mutations in *RYR1* and *CACNA1S* genes that primarily presents as a hypermetabolic state in response to volatile inhalational agents and the depolarizing neuromuscular blocking agent, succinylcholine. Medical conditions including central core disease, multi-minicore disease, central nuclear myopathy, and King-Denborough syndrome put patients at increased risk of MH. Intraoperatively, the earliest signs are a refractory rise in end-expired CO_2_ concentration, generalized muscle rigidity, increase in core temperature at the rate of 1–2°C every five minutes, rapid ATP consumption by skeletal muscle, and ultimately metabolic acidosis, life-threatening hyperkalemia, and myoglobinuria. The gold standard test for diagnosis is a caffeine-halothane contracture test performed on biopsied skeletal muscle fibers [1]. Intraoperatively, a Clinical Grading Scale for Malignant Hyperthermia is utilized which includes generalized rigidity in the absence of hypothermia, elevated creatinine kinase >10,000 IU after anesthetic without succinylcholine or >20,000 IU with succinylcholine, cola-colored urine, myoglobin in the urine >60 *μ*g/L, PaCO_2_ >60 mmHg with appropriately controlled ventilation, temperature increase >38.8°C, and cardiac arrhythmias [2]. Volatile anesthetics and succinylcholine must be avoided and either regional anesthesia or TIVA should be utilized in a patient who is suspected to be at risk for MH or with a prior MH history.

We describe the first case of an orthotopic liver transplant in an acutely decompensated patient with alcoholic cirrhosis (MELD 41), renal failure requiring CRRT, and a personal history of malignant hyperthermia as a child. A nontriggering anesthetic using TIVA with propofol and sufentanil infusions provided hemodynamic stability in this critically ill individual. Very few previous cases have described MH-susceptible patients undergoing OLTx and the majority of these involved intraoperative triggering in a previously unknown MH-sensitive patient necessitating the switch to TIVA during the procedure [3–5]. The only other case in which MH history was known prior to OLTx occurred in 1998 and the choice of anesthetic agents included continuous midazolam and fentanyl infusions [6].

Employing a propofol-based TIVA for OLTx presents unique challenges compared to using volatile anesthetics. The most common method of monitoring anesthetic depth during TIVA is with the BIS monitor. BIS levels have been reported to be lower in patients presenting for OLTx, particularly in patients with more severe liver disease [7, 8]. This is attributed to the acute illness and hepatic encephalopathy and was observed in our patient even at the low doses of propofol being used for sedation (BIS range 63–68 prior to initiation of TIVA). This raises the question of what BIS number should be used as a target for adequate depth of anesthesia during OLTx, or whether BIS is even an accurate monitor of anesthetic depth in this patient population.

Another challenge of using propofol as an anesthetic in OLTx relates to the altered pharmacokinetics and pharmacodynamics of propofol that may occur during the course of OLTx, as well as in the setting of hemorrhage and resuscitation. Liver transplantation involves large hemodynamic swings and can be accompanied by a large volume of blood loss. Taken together, these factors make inhalational general anesthesia the preferred anesthetic for these cases as large fluctuations in blood volume do not influence inhalational levels of anesthetic vapor. Interestingly, propofol requirements are reduced under target-controlled infusion-based TIVA, particularly during the anhepatic phase of OLTx [9–11], at least partially due to higher measured propofol blood concentrations compared to the target level [7, 11]. This is likely due to reduced clearance of propofol during the anhepatic phase. Additionally, several animal models and some human studies have shown that in the setting of massive hemorrhage, propofol levels increase due to a decrease in the volume of distribution of the central compartment and decreased clearance [12]. There also appears to be an increase in sensitivity to propofol both during hemorrhage and after resuscitation, which may be due in part to decreases in protein levels and an increase in the unbound fraction of propofol [12, 13]. Taken together, the many factors involved in OLTx including anhepatic and neohepatic phases, large volume blood loss, decreased circulating protein levels both pre-operatively in the setting of the decreased synthetic function of the native liver as well as intraoperatively secondary to hemorrhage, and decreased baseline BIS in critically ill patients with hepatic encephalopathy makes TIVA challenging in this patient population. Furthermore, many of the physiologic changes that are often observed during OLTx can resemble changes seen with malignant hyperthermia episodes including tachycardia, hyperkalemia, metabolic acidosis, and hypercarbia. This is further complicated by the absence of a definitive, intra-operative test to determine if a patient is experiencing an MH event and the fact that the treatment for an MH episode, dantrolene, can be hepatotoxic to an already marginally functioning liver or newly transplanted organ allograft [3]. Here, we report on a case where inhalational general anesthesia was contraindicated due to a presumed diagnosis of malignant hyperthermia and a successful TIVA for OLTx without signs of an MH episode in our patient.

## Figures and Tables

**Table 1:** Relevant intraoperative data.

Vitals	
Heart rate	42–75 beats per minute
Blood pressure	95/41–158/61 mmHg
Temperature	35.0–37.1°C

*Vasopressors*	
Norepinephrine	0.00–0.05 mcg/kg/min
Vasopressin	0.00–0.03 units/min

*Blood products*	
Packed red blood cells	5 units
Fresh frozen plasma	1 unit
Cryoprecipitate	20 units (5 units/bag)
Platelets	1 unit

*Ins/Outs*	
Crystalloid	5,300 mL
Cell saver	2,168 mL
Estimated blood loss	6,000 mL

## Data Availability

Not Applicable.

## Abbreviations

ASA: American society of anesthesiologists
BIS: Bispectral index
BMI: Body mass index
CO_2_: Carbon dioxide
CPR: Cardiopulmonary resuscitation
CRRT: Continuous renal replacement therapy
ICU: Intensive care unit
MAP: Mean arterial pressure
MELD: Model for end-stage liver disease
MH: Malignant hyperthermia
OLTx: Orthotopic liver transplant
OR: Operating room
TIVA: Total intravenous anesthetic
UNOS: United network for organ sharing

## Supplementary Materials

Supplementary Materials 1. CARE checklist for case reports. 2. Blank consent form.
