# Supplementary material for: Orthotopic Liver Transplantation in a Patient with Acutely Decompensated Liver Disease and Personal History of Malignant Hyperthermia
Source: Case Rep Anesthesiol. 2022 Sep 17;2022:4996977. doi: 10.1155/2022/4996977 (PMC9509212; doi:10.1155/2022/4996977)
Supplement: Supplementary Materials — 1. CARE checklist for case reports. 2. Blank consent form. [file 4996977.f1.zip › 4996977.f1/Townsend_CARE_Checklist.pdf]

CARE
case report guidelines

CARE Checklist of information to include when writing a case report

(cc) BY-NC-ND

Topic	Item	Checklist item description	Reported on Line
**Title**	**1**	The diagnosis or intervention of primary focus followed by the words "case report"	Page 1 Lines 1-2
**Key Words**	**2**	2 to 5 key words that identify diagnoses or interventions in this case report, including "case report"	Page 1 Line 34
**Abstract (no references)**	**3a**	Introduction: What is unique about this case and what does it add to the scientific literature?	Page 1 Lines 14-21
	**3b**	Main symptoms and/or important clinical findings	Page 1 Lines 22-23
	**3c**	The main diagnoses, therapeutic interventions, and outcomes	Page 1 Lines 23-26; 30-31
	**3d**	Conclusion—What is the main "take-away" lesson(s) from this case?	Page 1 Lines 27-32
**Introduction**	**4**	One or two paragraphs summarizing why this case is unique (**may include references**)	Page 1, 37-47; Page 2 lines 1-5
**Patient Information**	**5a**	De-identified patient specific information.	Page 2 Lines 8-19
	**5b**	Primary concerns and symptoms of the patient.	Page 2 lines 11-19; ~~27~~ 31
	**5c**	Medical, family, and psycho-social history including relevant genetic information	Page 2 9-10; 21-26
	**5d**	Relevant past interventions with outcomes	Page 2 lines 21-22
**Clinical Findings**	**6**	Describe significant physical examination (PE) and important clinical findings.	Page 2 lines 28-31; 36
**Timeline**	**7**	Historical and current information from this episode of care organized as a timeline	Page 2 lines 8-47, Page 3 1-7
**Diagnostic Assessment**	**8a**	Diagnostic testing (such as PE, laboratory testing, imaging, surveys).	Page 2 Lines 17-19
	**8b**	Diagnostic challenges (such as access to testing, financial, or cultural)	N/A
	**8c**	Diagnosis (including other diagnoses considered)	Page 2 Lines 11-14
	**8d**	Prognosis (such as staging in oncology) where applicable	Page 2 lines 13-14
**Therapeutic Intervention**	**9a**	Types of therapeutic intervention (such as pharmacologic, surgical, preventive, self-care)	Page 2 Lines 37-47, [illegible]
	**9b**	Administration of therapeutic intervention (such as dosage, strength, duration)	Page 2 Lines 37-47 Page 3; 1-7
	**9c**	Changes in therapeutic intervention (with rationale)	N/A
**Follow-up and Outcomes**	**10a**	Clinician and patient-assessed outcomes (if available)	Page 3 lines 4-7
	**10b**	Important follow-up diagnostic and other test results	N/A
	**10c**	Intervention adherence and tolerability (How was this assessed?)	Page 2 Lines 43; Page 3 Line 7
	**10d**	Adverse and unanticipated events	N/A
**Discussion**	**11a**	A scientific discussion of the strengths AND limitations associated with this case report	Page 3 lines 25-46; Page 4 1-19
	**11b**	Discussion of the relevant medical literature **with references**.	Page 3 lines 10-46; Page 4 1-19
	**11c**	The scientific rationale for any conclusions (including assessment of possible causes)	Page 3 lines 33-46 Page 4 1-12
	**11d**	The primary "take-away" lessons of this case report (without references) in a one paragraph conclusion	Page 4 lines 17-19
**Patient Perspective**	**12**	The patient should share their perspective in one to two paragraphs on the treatment(s) they received	N/A - Anesthetized
**Informed Consent**	**13**	Did the patient give informed consent? Please provide if requested	Yes ☒ No ☐
